# Rapid MALDI-TOF Mass Spectrometry Identification of the Chalkbrood Pathogen *Ascosphaera apis*

**DOI:** 10.3390/jof12050311

**Published:** 2026-04-23

**Authors:** Barbara Hočevar, Darja Kušar, Igor Gruntar, Cene Gostinčar, Irena Zdovc

**Affiliations:** 1 Institute of Pathology, Forensic Veterinary Medicine, Wildlife, Bees and Aquaculture, Veterinary Faculty, University of Ljubljana, Gerbičeva 60, 1000 Ljubljana, Slovenia; 2 Institute of Microbiology and Parasitology, Veterinary Faculty, University of Ljubljana, Gerbičeva 60, 1000 Ljubljana, Slovenia; darja.kusar@vf.uni-lj.si (D.K.); igor.gruntar@vf.uni-lj.si (I.G.); irena.zdovc@vf.uni-lj.si (I.Z.); 3 Chair of Molecular Genetics and Biology of Microorganisms, Department of Biology, Biotechnical Faculty, University of Ljubljana, Jamnikarjeva 101, 1000 Ljubljana, Slovenia; cene.gostincar@bf.uni-lj.si

**Keywords:** *Ascosphaera apis*, matrix-assisted laser desorption/ionization time-of-flight mass spectrometry (MALDI-TOF MS), in-house library, species identification, whole-genome sequencing (WGS), honeybee fungal diseases

## Abstract

*Ascosphaera apis* is a fungal pathogen of honeybee larvae and the primary cause of chalkbrood disease, which weakens bee colonies, impairing their ability to function effectively and making them more susceptible to other pathogens and environmental stressors. This study aimed to develop and validate an in-house matrix-assisted laser desorption/ionization time-of-flight mass spectrometry (MALDI-TOF MS) spectral library for *A. apis*. A new MALDI-TOF MS library was constructed using reference *Ascosphaera* species and validated through whole-genome-based confirmation of 31 clinical isolates of *A. apis*. Three different protein extraction methods were tested and compared: liquid cultivation, formic acid–ethanol extraction and extended direct transfer. Our findings demonstrate that MALDI-TOF MS is a rapid and reliable tool for identifying *A. apis* under the tested laboratory conditions and within the analyzed strain set, with no misidentifications observed for the liquid cultivation and formic acid–ethanol extraction methods. The extended direct mycelium transfer method was slightly less effective but still showed a high sensitivity of 83.9%. This study provides a foundation for improving diagnostic approaches in the management of honeybee fungal diseases.

## 1. Introduction

*Ascosphaera* is a genus of fungi comprising 28 described species closely associated with bees, occurring either as entomopathogens or as saprotrophic species within the bee nest environment [1]. *Ascosphaera apis* is the best-studied species of the genus due to its role as the primary etiological agent of chalkbrood disease [2]. Its economic significance stems from its impact on populations of commercially important pollinators. Chalkbrood disease rarely causes bee colony death but can significantly reduce colony strength, productivity and honey production [3], representing a major concern for beekeepers. Clinical signs of chalkbrood disease are characterized by fluffy white fungal growth on bee larvae, which eventually desiccate and form white, grey or black “mummies” [4]. These mummified larvae can be easily removed from brood cells, serving as a differential diagnostic feature that distinguishes chalkbrood from stonebrood, which is caused by *Aspergillus* species as described by Jensen et al. [5]. Some studies suggest that *Ascosphaera major* can be associated with clinical symptoms similar to those caused by *A. apis* [1], complicating species identification based on clinical signs alone. Asexual reproduction has not been described for *A. apis*, which reproduces exclusively sexually; when opposite mating types are present in the same culture, sexual reproduction results in spore formation that macroscopically appear as dark lines at the interface between the two mating types [6]. Particularly in mixed cultures, this can further complicate morphological differentiation between *Ascosphaera* species as their reproductive structures are highly similar, further limiting the reliability of traditional diagnostic methods [1,7,8]. Therefore, the most reliable methods for identifying *Ascosphaera* fungi are molecular techniques such as polymerase chain reaction (PCR) and DNA sequencing.

In recent years, matrix-assisted laser desorption/ionization time-of-flight mass spectrometry (MALDI-TOF MS) has become a rapid and reliable tool for fungal identification. The method is primarily based on analyzing ribosomal protein profiles with molecular masses ranging from 2000 to 20,000 Da [9,10,11]. A representative protein profile spectrum of the analyzed microorganism is constructed and compared with existing reference libraries. The first application of MALDI-TOF MS to fungi was reported by Welham et al. [12], in which fungal spores were used as the target material. Subsequent developments focused on the use of mycelium and the optimization of protein extraction protocols. Filamentous fungi possess a rigid cell wall that requires disruption for effective protein extraction, necessitating the use of specialized protocols tailored to fungal structural characteristics. Three extraction methods for filamentous fungi are proposed by the MS instrument manufacturer Bruker Daltonics: liquid cultivation, formic acid–ethanol extraction and extended direct transfer [13]. Although the use of MALDI-TOF MS for fungal identification in laboratories has been increasing, its application to filamentous fungi remains less widespread than for bacteria and yeasts. This is primarily due to the more time-consuming sample preparation required for filamentous fungi, as well as the relatively sparse representation of these species in available mass spectral libraries.

Currently, the Bruker MALDI-TOF MS database lacks protein spectra and reference data for *A. apis* and other *Ascosphaera* species, limiting the applicability of this method for the identification of bee-associated filamentous fungi. Despite the increasing use of MALDI-TOF MS in mycology, studies focusing on entomopathogenic fungi, particularly those associated with pollinators, remain scarce.

In this study, we aimed to create and validate an in-house MALDI-TOF MS library specifically for *A. apis* and related species. Species identification was previously confirmed by whole-genome sequencing (WGS) of 31 field *A. apis* isolates and seven reference *Ascosphaera* strains (*A. apis*, *A. atra*, *A. duoformis*, *A. flava*, *A. larvis*, *A. major* and *A. proliperda*). Our objective was to demonstrate the effectiveness of MALDI-TOF MS using the new *Ascosphaera* spectral library for rapid and reliable identification of *A. apis* directly from cultured isolates, providing a practical alternative to molecular and morphological methods in apicultural diagnostics.

This work provides the first MALDI-TOF MS-based approach for the identification of *A. apis* and contributes to expanding the applicability of mass spectrometry in fungal diagnostics, particularly for underrepresented filamentous fungi, and underscores the continued evolution of MALDI-TOF MS as an essential tool for identifying the previously challenging fungal species.

## 2. Materials and Methods

### 2.1. Reference Strains and Clinical Isolates

Four *A. apis* reference strains and six reference strains from other *Ascosphaera* species—sourced from the Agricultural Research Service (ARSEF, USDA)—were selected as well-characterized isolates from curated collections to represent the investigated genus’ diversity and to serve as the foundation for the new MALDI-TOF MS database development (Table 1). Local *A. apis* isolates used for validation originated from clinical samples (*n* = 31), collected from mummified honeybee larvae in Slovenian apiaries exhibiting symptoms of chalkbrood, and were confirmed as *A. apis* by whole-genome sequencing (WGS; Section 2.4, Table S1).

### 2.2. Cultivation of Ascosphaera Strains and MALDI-TOF MS Identification

**Figure 1:**
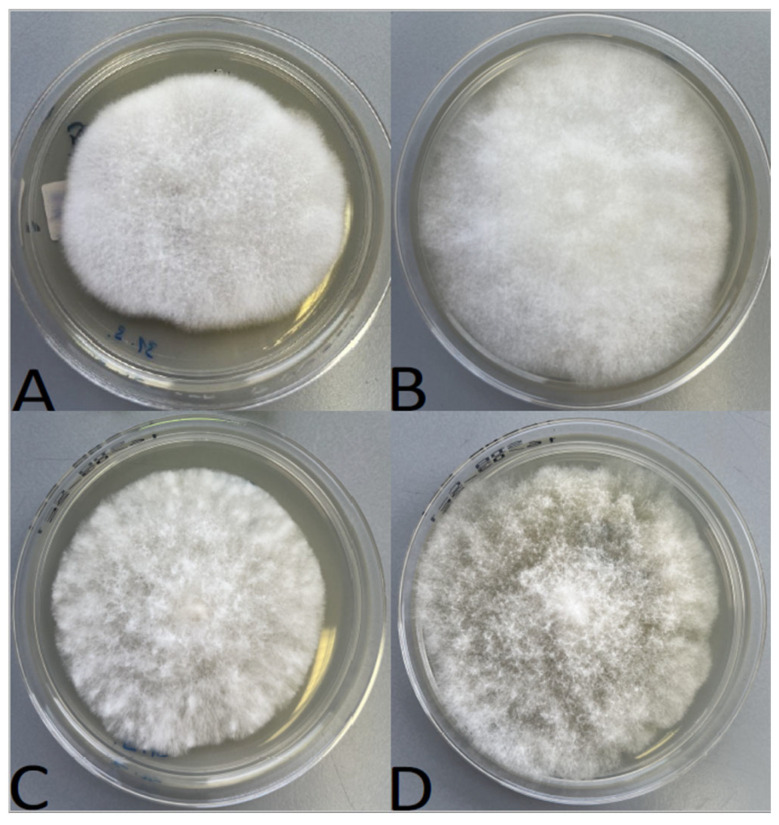
Mycelia of *Ascosphaera* fungi growing on Sabouraud Dextrose Agar (SDA) plates: (**A**) *A. apis* (ARSEF 7405), (**B**) *A. larvis* (ARSEF 7945), (**C**) *A. proliperda* (ARSEF 695) and (**D**) *A. major* (ARSEF 694).

Honeybee larvae showing clinical signs of chalkbrood were surface disinfected with a diluted (1:9, *v*/*v*) sodium hypochlorite solution (household bleach) for 10 min, rinsed twice with sterile distilled water for 2 min and aseptically cut into pieces, which were laid on Sabouraud Dextrose Agar (SDA; Biolife, Milano, Italy) plates and incubated at 30 °C [5]. Once initial mycelial growth was observed, cultures were subcultured to obtain pure isolates and single mating types to prevent sporulation. The reference strains and clinical isolates were then cultured on SDA plates and incubated at 30 °C for 2–4 days to promote mycelial growth. Colony morphology was documented (Figure 1) and isolates were identified using MALDI-TOF MS (Microflex LT System; Bruker Daltonics, Bremen, Germany). To obtain suitable protein extracts for MALDI-TOF MS identification, fungal cultures were prepared using all three protein extraction procedures, as described below.

#### 2.2.1. Liquid Cultivation Method

For the identification of isolates, we followed the manufacturer’s protocol for liquid cultivation sample preparation [13], with minor modifications to accommodate the genus-specific characteristics of *Ascosphaera*. After a pure culture was obtained on SDA plates, the culture was transferred to Sabouraud Dextrose Broth (Bio Merieux, Marcy-l’Étoile, France) and incubated on a rotary shaker (IKS 4000 i control, IKA, Staufen, Germany) at 140 rpm and 30 °C for 48 h. The mycelium was collected using a sterile loop, transferred into an Eppendorf tube, and centrifuged for 2 min at 16,500 *g*; the supernatant was discarded.

The mycelial pellet was resuspended in 1 mL of sterile distilled water and vortexed for 1 min. This was followed by centrifugation for 2 min at 16,500 *g*. The supernatant was carefully removed until 300 µL remained, after which 900 µL of absolute ethanol was added and the mixture was vortexed again. The sample was centrifuged for 2 min at 16,500 *g* and the supernatant was removed. The pellet was completely dried at 37 °C before the addition of 70% formic acid (Sigma-Aldrich by Merck, Darmstadt, Germany) in proportion to the pellet size (10–20 µL for small pellets, up to 100 µL for large pellets). An equal volume of acetonitrile (Fluka Analytical, Seelze, Germany) was added and the mixture was vortexed. The sample was then centrifuged for 2 min at 16,500 *g* and 1 µL of the resulting supernatant was applied to a MALDI-TOF plate and allowed to dry. Finally, 1 µL of α-cyano-4-hydroxycinnamic acid solution (HCCA matrix; Sigma-Aldrich by Merck, Germany), prepared according to the manufacturer’s recommendations, was added and air-dried at room temperature before analysis.

This protocol was applied to both *Ascosphaera* (reference strains and clinical isolates) and *Aspergillus* species. The latter were included as *Aspergillus* species are well represented in MALDI-TOF MS databases and perform reliably [14]. In our analyses, *A. flavus* served as a MALDI-TOF MS quality control, ensuring accurate validation of instrument performance, while *A. flavus*, *A. fumigatus* and *A. niger* were used as an outgroup in the constructed phylogenetic tree (Section 2.4, Figure S1).

#### 2.2.2. Formic Acid–Ethanol Extraction Method

To assess the feasibility of a more rapid sample preparation method, a formic acid–ethanol protein extraction method (also known as the ethanol extraction method) was tested on clinical samples alongside the standard liquid cultivation method. The mycelium of *A. apis* was collected directly from the edge of actively growing colonies on solid media using a sterile inoculation loop. Approximately 300 µL of 70% ethanol was added to the collected mycelium, and the sample was vortexed for 1 min to ensure a homogeneous suspension. The sample was then centrifuged for 2 min at 16,500 *g*, the supernatant was discarded, and the pellet was air-dried. Next, 25–50 µL of 70% formic acid and an equal volume of acetonitrile were added to the pellet. The sample was vortexed for 1 min and centrifuged for 2 min at 16,500 *g*. After extraction, 1 µL of the resulting supernatant was applied to the MALDI target plate and allowed to air-dry at room temperature. Once dry, the spot was overlaid with 1 µL of the HCCA matrix. The treated sample was dried again and analyzed using the MALDI-TOF MS system.

#### 2.2.3. Extended Direct Transfer Method

According to Bruker’s guidelines, we also evaluated the extended direct mycelium transfer method on clinical samples. Briefly, 1 µL of formic acid was applied to a MALDI-TOF MS target plate. A sterile toothpick was dipped into the formic acid droplet and then used to transfer young mycelium from the peripheral region of a fungal colony grown on SDA plates to the formic acid droplet on the target plate. The spot was allowed to air-dry, after which 1 µL of HCCA matrix solution was added. After drying again, the target plate was inserted into the MALDI-TOF MS instrument for analysis.

### 2.3. Validation of the In-House Library and Identification of Isolates

The spectra were acquired and generated using a Bruker Microflex LT/SH MALDI-TOF MS instrument (Bruker Daltonics, Bremen, Germany) in positive linear mode across a mass range (*m*/*z*) of 2000–20,000 Da, operated via FlexControl Compass flexSeries 1.4 software. Spectra analysis, comparison, and identification were managed using MBT Compass HT software version 5.1.300 and relevant Main Spectrum Profile (MSP) sources or libraries (Filamentous Fungi V. 2023 and our in-house *Ascosphaera* libraries).

Reference spectra (MSPs) were generated using four *A. apis* reference strains and one strain each from six other *Ascosphaera* species (Table 1). Reference MSPs were generated from four independent subcultures of each reference strain, with one MALDI-TOF MS analysis per subculture using Bruker Biotyper software (Maldi BioTyper Compas Explorer 4.1) according to the manufacturer’s instructions. The newly developed in-house library was validated by analyzing spectra derived from 31 clinical *A. apis* isolates, obtained from chalkbrood-infected honeybee larvae collected from various apiaries (Section 2.1, Table S1). Identification accuracy was evaluated based on the log score values: scores ≥ 2.0 indicate high-confidence species-level identification, scores 1.70–1.99 indicate low-confidence genus-level identification, and scores < 1.70 are considered unreliable for conclusive identification (Bruker Daltonics, Germany).

### 2.4. WGS of Reference Strains and Clinical Isolates

After culturing the clinical isolates on SDA plates and subsequently in Sabouraud Dextrose Broth for the liquid extraction protocol, a portion of the resulting mycelium was stored at −70 °C. This was used for DNA extraction and WGS for molecular confirmation of the species to complement the MALDI-TOF MS identification. Seven reference *Ascosphaera* strains (*A. apis* ARSEF 7405, *A. atra*, *A. duoformis*, *A. flava*, *A. larvis*, *A. major* and *A. proliperda*; Table 1 and Table S1) and all clinical *A. apis* isolates (*n* = 31; Table S1) were subjected to WGS to enable construction of a phylogenetic tree of *Ascosphaera* spp. (Figure S1), also incorporating other publicly available *Ascosphaera* genomes (*A. acerosa* GCA_024244165.1, *A. aggregata* GCA_024244195.1, *A. apis* ARSEF 7405 GCA_001636715.1, *A. atra* GCA_024244595.1 and *A. pollenicola* GCA_024244615.1). *Aspergillus* species (clinical isolates and reference genomes of *A. flavus*, *A. fumigatus* and *A. niger*) were included as an outgroup (Table S1).

For the extraction of DNA from mycelia of *Ascosphaera* and *Aspergillus* isolates, DNeasy Plant Pro Kit (Qiagen, Hilden, Germany) was used according to the manufacturer’s instructions with some modifications. In brief, 200–400 mg of fungal mycelia (approximately one-fifth to one-third of 2 mL tube) was used. After the mycelia were transferred to the tissue disruption tubes and solution CD1 was added, samples were incubated for 10 min at 65 °C to increase DNA yield. Samples were homogenized in two disruption periods of 30 s at 3000 rpm, with a 2 min cooling pause, using the MagNA Lyser Instrument (Roche, Basel, Switzerland). After homogenization, 4 µL of RNase (Qiagen, Germany) was added to each sample for RNA degradation at room temperature for 10 min, and the extraction was then continued as recommended.

Sequencing libraries were prepared using the Novogene NGS Library Preparation Kit and paired-end (2 × 150 bp) sequencing was performed on an Illumina NovaSeq X Plus Series (Illumina, San Diego, CA, USA) to a minimum output of 5 Gbp. The raw sequencing data was processed with fastp 0.23.4 with automatic trimming of any remaining adapter sequences and ends with a mean Phred quality score below 20 in 3 bp windows [15]. Any reads shorter than 50 bp after trimming were discarded. Duplicate reads were removed using the accuracy parameter value of 5. Assembly was performed using SPAdes genome assembler v4.0.0 with the option “--isolate” [16]. The quality of assemblies was assessed with QUAST v5.2.0 and BUSCO v5.8.3, using the “onygenales_odb12” database [17,18]. Benchmarking Universal Single-Copy Orthologs (BUSCO) present in all analyzed genomes as single-copy genes were aligned with MAFFT v7.505 using the option “--auto” and the alignments were concatenated into a superalignment with AMAS.py script [19]. This was used to estimate a phylogenetic tree with IQ-TREE v2.3.6 using the estimation of the best substitution model and approximate likelihood-ratio test (aLRT) for branches (“-m TEST --alrt 1000”) [20]. The sequencing data generated in this study were deposited in the NCBI Sequence Read Archive (SRA) database under the BioProject accession number PRJNA1390123; a complete list of the obtained genomes is available in Table S1.

### 2.5. Statistical Analysis

Statistical analysis was performed using R software, v4.3.2 [21]. Differences in identification outcomes among extraction methods were evaluated using Cochran’s Q test for paired binary data, followed by NcNemar’s test for pairwise comparisons. Statistical significance was set at *p* < 0.05.

## 3. Results

**Figure 2:**
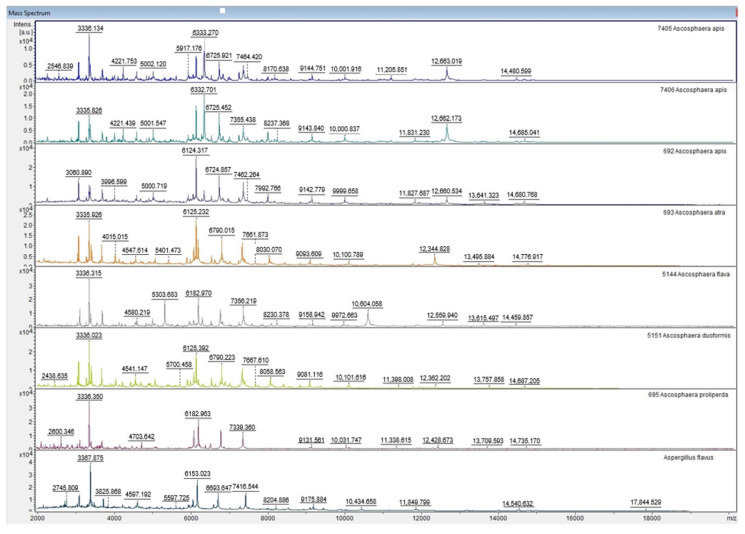
Selected MALDI TOF MS spectra of *Ascosphaera* fungi obtained by the liquid extraction method, including reference strains of *A. apis* (ARSEF 7405, ARSEF 7406, ARSEF 692), *A. atra* (ARSEF 693), *A. flava* (ARSEF 5144), *A. duoformis* (ARSEF 5151) and *A. proliperda* (ARSEF 695); *Aspergillus flavus* is included as a control species.

To develop an internal database for the identification of *A. apis* by MALDI-TOF MS, we generated reference MSPs from *Ascosphaera* reference strains (Table 1) and compiled them into a corresponding in-house MSP library. For library validation, we used results obtained from testing both clinical and reference strains of *A. apis*, as well as some other species within the same genus. In addition, we assessed the reliability of the identification results using three different extraction methods. Upon visual inspection, the MALDI-TOF MS protein spectra of different *Ascosphaera* species were highly similar (Figure 2). However, specific reproducible signals at approximately *m*/*z* 10.000 and 12,660 were consistently detected for *A. apis*, being absent in the spectra of all other *Ascosphaera* species analysed.

In this study, all identifications with a log score > 1.70 were considered positive. As none of the six reference strains representing other *Ascosphaera* species (Table 1) were misidentified as *A. apis*, no misidentifications were observed within the tested dataset (Table 2).

Using both the liquid cultivation method and the formic acid–ethanol extraction method, and employing the constructed in-house reference library, all clinical isolates (*n* = 31), which were also confirmed as *A. apis* by WGS (Figure S1), were correctly identified as *A. apis*. No misidentifications occurred. The lowest identification reliability was observed with the extended direct transfer method. In this case, 45.2% of isolates yielded high-confidence identifications, 38.7% yielded low-confidence identifications and 16.1% were not reliably identified, resulting in 83.9% sensitivity, while no misidentifications were observed among the analyzed isolates (Table 2).

A Cochran’s Q test showed a statistically significant difference in identification outcomes among the three extraction methods (Cochran’s Q = 10.0, df = 2, *p* = 0.0067). Pairwise comparison demonstrated that the extended direct transfer method showed significantly lower identification success compared with the formic acid–ethanol extraction method (McNemar’s χ^2^ = 5.0, df = 1, *p* = 0.025); the liquid cultivation and formic acid–ethanol extraction methods produced identical identification outcomes.

## 4. Discussion

Despite the availability of advanced diagnostic techniques, identification of filamentous fungi in many laboratories still predominantly relies on morphological and microscopic examination. These methods require highly trained personnel and are inherently subjective, potentially leading to inconsistencies in identification. In contrast, molecular approaches offer high accuracy but are often limited by time-consuming and technically demanding protocols and the need for advanced bioinformatics expertise. Therefore, it is essential to integrate rapid, reliable and accurate methods, such as MALDI-TOF MS, into routine diagnostic workflows. Although the application of MALDI-TOF MS to filamentous fungi has increased in recent years, available fungal libraries remain limited, especially for species causing bee diseases.

*Ascosphaera* species are currently not included in the in-built Bruker database, which significantly hinders identification and differentiation within this genus. Morphological features, both macroscopic on culture media and microscopic, are often difficult to distinguish among *Ascosphaera* species, as differentiation relies mainly on the characteristics of reproductive structures, which frequently overlap between species. Additionally, several *Ascosphaera* species do not sporulate in vitro, and because some species are heterothallic, sporulation does not occur when only one mating type is present [1,7,8]. Consequently, morphology-based identification of *Ascosphaera* species is often unreliable and subjective. This highlights the importance of expanding MALDI-TOF MS spectral libraries to include ecologically and clinically relevant fungal genera, such as *Ascosphaera*, to improve diagnostic precision. Considerable uncertainty remains regarding the optimal approach to constructing an in-house reference library, as many studies use different methods, versions of methods or criteria for library creation [9,11,22]. In our study, we constructed an in-house library using four *A. apis* reference strains and one reference strain for each of the six additional *Ascosphaera* species. For each reference strain, four independent subcultures were used to generate the reference MSP, as biological replication has been shown to contribute more to identification performance than further increases in the number of technical replicates [23]. The liquid extraction protocol was used to obtain the reference spectra, as this extraction method was also used in the creation of the commercial MBT Filamentous Fungi Library. Although the mass spectra of different *Ascosphaera* species appeared visually similar, with species-specific individual signals, MALDI TOF MS Biotyper identification relies on algorithm-based comparison of the entire spectrum.

The cell walls of *A. apis* spores contain the pigment melanin [24]. Melanin and other pigments may interfere with MALDI-TOF MS by competing with the matrix for photon absorption, leading to suppressed ionization and reduced peak detection. This distorts mass spectra and compromises the reliability of identification [25]. According to the manufacturer, liquid cultivation method minimizes the impact of culture conditions on the resulting mass spectra. It helps to standardize the physiological state of the organism and promotes the formation of uniform mycelium. Moreover, our observations confirmed that *A. apis* does not sporulate during liquid cultivation, consistent with earlier reports showing that growth in liquid media suppresses sporulation [26], which contributes to the generation of a reproducible and robust spectral library.

For all extraction methods, mycelium was collected as soon as adequate mycelium development was observed, which occurred after two days of growth. Although some studies suggest that variations in incubation temperature, culture medium and mycelium age do not significantly affect identification success [27], other studies have shown that cultivation parameters, including mycelial age, incubation temperature and culture medium, can affect spectral reproducibility and peak composition [14,28,29]. To minimize variability and ensure reproducible, high-quality spectra across isolates, all of them were cultured under the same standardized conditions of medium, temperature and incubation time, and these conditions were maintained consistently for each extraction method before the MALDI-TOF MS analysis.

When comparing different protein extraction methods, Honsig et al. [30] reported successful identification in 86.4% (log score ≥ 1.7) of filamentous fungal isolates using the liquid extraction method. In our study, the liquid extraction method correctly identified all *A. apis* clinical isolates, with high log score values of ≥2.0 and full concordance with sequencing results, confirming the robustness and reliability of this protocol in our setting.

The formic acid–ethanol extraction method, developed as a faster alternative, has shown variable performance with other fungi in previous reports, with identification success ranging from 30.8 to 59.4% [14] to 84.2% at log score ≥ 1.7 [30]. In our study, this method also correctly identified all *A. apis* isolates at log score ≥ 2.0, demonstrating that for *A. apis*, it can serve as an effective and time-efficient option for accurate identification at species level. Such a high level of specificity is probably also due to the fact that fungi of the genus *Ascosphaera* have a uniquely distinct protein profile, which differs significantly from those of other known fungi in the current library, making misidentification less likely.

The extended direct transfer method, while being the fastest and most convenient, has consistently been associated with reduced identification accuracy, particularly for filamentous fungi with tough cell walls. Previous studies have reported success rates at log scores ≥ 1.7 as low as 16.7–53.9% [14] and 72.8% [30]. In our study, the extended direct method yielded an overall identification success rate of 83.9%, with 45.2% of isolates identified with high confidence (log score ≥ 2.0), 38.7% with low confidence (log score 1.7–1.99), and 16.1% remaining unidentified (log score < 1.7). These results confirm that although the extended direct transfer method did not produce misidentifications, its lower proportion of high-confidence identifications requires careful application; the observed differences between extraction methods are supported by statistical analysis and should be interpreted within the scope of the tested dataset.

Distinct and reproducible signals at approximately *m*/*z* 10,000 and 12,660 were observed in *A. apis* spectra under the tested conditions and were not detected in the other *Ascosphaera* species included in this study. However, these observations are based on visual inspection of spectra and were not further validated in this study.

All three extraction methods evaluated in this study were performed on *Ascosphaera* species grown on SDA, although some previous studies have reported that SDA is inferior to other culture media for MALDI-TOF MS identification of filamentous fungi when the formic acid–ethanol extraction method and extended direct transfer method are applied [14]. For *A. apis*, SDA performed well with the formic acid–ethanol extraction method. However, for the extended direct transfer method, testing additional culture media would be necessary to determine whether the lower identification success observed can be attributed, at least in part, to medium-dependent effects.

In cases of sporulation, the liquid culture extraction method is preferred for generating high-quality, reproducible MALDI-TOF MS spectra. However, when this method is used, isolates must first be recovered from the clinical samples and sub-cultured on solid media to obtain a pure culture prior to liquid cultivation, which prolongs identification time. Therefore, if only one mating type is present, or sporulation has not yet occurred, the extended direct transfer method is an appropriate first-line extraction approach, as it yielded a relatively high rate of correct *A. apis* identification in our study. In cases where this method fails to produce a confident identification, the formic acid–ethanol extraction method can be applied as a more reliable alternative.

The library was validated using 31 clinical *A. apis* isolates, with species confirmed by WGS, ensuring accurate reference identification for library construction. Accordingly, this study represents a controlled validation using isolates with known identities rather than a prospective diagnostic evaluation.

We exclusively sampled mummified honeybee larvae exhibiting clinical signs of chalkbrood disease from Slovenian apiaries, where only *Apis mellifera carnica* is legally bred. According to the existing literature, the chalkbrood-like symptoms in honeybees are predominantly caused by *A. apis* and, less commonly, *A. major* [1]. The pathogenic role of *A. major* has not yet been confirmed, suggesting that it may act as a secondary colonizer rather than a true etiological agent. In our sampling, *A. major* was not detected.

It should be noted, however, that extending the sampling scope to other insects, pollinators, and geographic regions, together with inclusion of additional *Ascosphaera* reference strains, will be essential for developing a more comprehensive MALDI-TOF MS library capable of reliably distinguishing closely related species and supporting broader diagnostic application.

## 5. Conclusions

This study demonstrates that MALDI-TOF MS is a rapid and reliable tool for identifying *Ascosphaera* species, addressing the limitations associated with classical and molecular methods. The high accuracy of *A. apis* identification using the constructed in-house *Ascosphaera* library highlights the potential for integrating MALDI-TOF MS into routine honeybee disease diagnostics, reducing reliance on culture-based methods alone. The ability to use the formic acid–ethanol extraction method from solid media further supports the practicality of MALDI-TOF MS for routine diagnostic use. Although both the liquid culture and formic acid–ethanol extraction methods produced higher-quality spectra, the extended direct transfer method still provided sufficient resolution for accurate identification of *A. apis*, offering a fast and very convenient alternative. The current in-house MALDI-TOF MS library includes seven species of the genus *Ascosphaera* and was designed primarily for reliable identification of *A. apis*. Accordingly, this study does not aim to establish comprehensive genus-level discriminatory performance. Expanding the library to include additional species would enhance identification capabilities for fungi within this genus. Future studies should also explore environmental and geographic variations in *Ascosphaera* spectra to better assess species variability.

## Figures and Tables

**Table 1:** *Ascosphaera* reference species obtained from ARSEF (USDA) used for MALDI-TOF MS database development. Strains subjected to genome sequencing (Section 2.4) are marked with an asterisk and their metadata are available in Table S1.

Ascosphaera Species	ARSEF Number	Origin
*A. apis*	7405 *	USA, Texas
*A. apis*	7406	USA, Texas
*A. apis*	691	USA, Oregon
*A. apis*	692	USA, Oregon
*A. atra*	693 *	USA, Oregon
*A. major*	694 *	Denmark
*A. proliperda*	695 *	Denmark
*A. larvis*	7945 *	Canada, Saskatchewan
*A. flava*	5144 *	Australia, Western Australia
*A. duoformis*	5151 *	/

**Table 2:** Comparison of MALDI-TOF MS identification results for Ascosphaera apis isolates using three protein extraction methods, including log score distribution, sensitivity and specificity within the analyzed dataset.

	Extraction Method		
Identification Result	Liquid Cultivation Method	Formic Acid–Ethanol Extraction Method	Extended Direct Transfer Method
High confidence (log ≥ 2.0)	31/31 (100%)	31/31 (100%)	14/31 (45.2%)
Low confidence (log 1.7–1.99)	0/31 (0%)	0/31 (0%)	12/31 (38.7%)
Not identified (log < 1.7)	0/31 (0%)	0/31 (0%)	5/31 (16.1%)
Sensitivity	100%	100%	83.9%
Specificity	100%	100%	100%

## Data Availability

The original contributions presented in this study are included in the article/Supplementary Material. The raw data supporting the conclusions of this article will be made available by the authors on request.

## Supplementary Materials

The following supporting information can be downloaded at https://www.mdpi.com/article/10.3390/jof12050311/s1, Table S1. Metadata of *Ascosphaera* reference strains, clinical isolates and genomes used in the study. Three *Aspergillus* species are also included. Figure S1. Phylogenetic tree based on a superalignment of 1394 BUSCOs (Benchmarking Universal Single-Copy Orthologs) present as single-copy genes in all included *Ascosphaera* and *Aspergillus* genomes. The sequences were aligned with MAFFT v7.505 and the tree was calculated using IQ-TREE v2.3.6 with automatic selection of the best-fitting substitution model. We obtained the gene sequences by sequencing, assembling and analyzing the genomes of 31 *Ascosphaera apis* environmental isolates obtained from clinical samples and seven *Ascosphaera* type strains, and also included publicly available genomes from five *Ascosphaera* type strains (Table S1). In addition, genomes of three *Aspergillus* isolates from clinical samples and three publicly available *Aspergillus* genomes were included (Table S1) as an outgroup. The scale bar for branch lengths represents the number of substitutions per site.
