# Supplementary material for: Rapid MALDI-TOF Mass Spectrometry Identification of the Chalkbrood Pathogen Ascosphaera apis
Source: J Fungi (Basel). 2026 Apr 23;12(5):311. doi: 10.3390/jof12050311 (PMC13208865; doi:10.3390/jof12050311)
Supplement: Supplementary file 1 [file jof-12-00311-s001.zip › jof-4251818-supplementary/Hocevar et al_Table_S1.pdf]

Table S1. Metadata of *Ascosphaera* reference strains, clinical isolates and genomes used in the study. Three *Aspergillus* species are also included.

***Ascosphaera* species**	**Strain/isolate designation**	**Origin**	**Genome / SRA run accession number**
Reference strains			
A. apis	ARSEF 7405 (AA32)	USA, Texas	SRR36484481*
A. apis	ARSEF 7406	USA, Texas	/
A. apis	ARSEF 691	USA, Oregon	/
A. apis	ARSEF 692	USA, Oregon	/
A. atra	ARSEF 693 (AA34)	USA, Oregon	SRR36484480*
A. duoformis	ARSEF 5151 (AA35)	/	SRR36484479*
A. flava	ARSEF 5144 (AA36)	Australia, Western Australia	SRR36484478*
A. larvis	ARSEF 7945 (AA37)	Canada, Saskatchewan	SRR36484477*
A. major	ARSEF 694 (AA38)	Denmark	SRR36484476*
A. proliperda	ARSEF 695 (AA39)	Denmark	SRR36484475*
Clinical isolates			
A. apis	AA1	Slovenia, apiary A	SRR36484506*
A. apis	AA2	Slovenia, apiary B	SRR36484505*
A. apis	AA3	Slovenia, apiary C	SRR36484494*
A. apis	AA4	Slovenia, apiary VF/P10	SRR36484483*
A. apis	AA5	Slovenia, apiary VF/P15/I/H1	SRR36484472*
A. apis	AA6	Slovenia, apiary VF/P15/I/H2	SRR36484470*
A. apis	AA7	Slovenia, apiary VF/P15/I/H3	SRR36484469*
A. apis	AA8	Slovenia, apiary VF/P15/II/H1	SRR36484468*
A. apis	AA9	Slovenia, apiary VF/P15/II/H2	SRR36484467*
A. apis	AA10	Slovenia, apiary VF/P15/II/H3	SRR36484466*
A. apis	AA11	Slovenia, apiary VF/P15/III/H1	SRR36484504*
A. apis	AA12	Slovenia, apiary VF/P15/III/H2	SRR36484503*
A. apis	AA13	Slovenia, apiary VF/P15/III/H3	SRR36484502*
A. apis	AA14	Slovenia, apiary D/P6/I/H1	SRR36484501*
A. apis	AA15	Slovenia, apiary D/P6/I/H2	SRR36484500*
A. apis	AA16	Slovenia, apiary D/P6/I/H3	SRR36484499*
A. apis	AA17	Slovenia, apiary D/P6/II/H1	SRR36484498*
A. apis	AA18	Slovenia, apiary D/P6/II/H2	SRR36484497*
A. apis	AA19	Slovenia, apiary D/P6/II/H3	SRR36484496*
A. apis	AA20	Slovenia, apiary D/P6/III/H1	SRR36484495*
A. apis	AA21	Slovenia, apiary D/P6/III/H2	SRR36484493*
A. apis	AA22	Slovenia, apiary D/P6/III/H3	SRR36484492*
A. apis	AA23	Slovenia, apiary D/P22/I/H1	SRR36484491*
A. apis	AA24	Slovenia, apiary D/P22/I/H2	SRR36484490*
A. apis	AA25	Slovenia, apiary D/P22/I/H3	SRR36484489*
A. apis	AA26	Slovenia, apiary D/P22/II/H1	SRR36484488*
A. apis	AA27	Slovenia, apiary D/P22/II/H2	SRR36484487*
A. apis	AA28	Slovenia, apiary D/P22/II/H3	SRR36484486*
A. apis	AA29	Slovenia, apiary D/P22/III/H1	SRR36484485*
A. apis	AA30	Slovenia, apiary D/P22/III/H2	SRR36484484*
A. apis	AA31	Slovenia, apiary D/P22/III/H3	SRR36484482*
Reference genomes			
A. acerosa	ATCC 201316	Canada, Alberta	GCA_024244165.1
A. aggregata	USDA-ARS PIBMSR Wild2	USA, Utah	GCA_024244195.1

A. apis	ARSEF 7405	USA, Texas	GCA_001636715.1
A. atra	ARSEF 5147	Australia, Western Australia	GCA_024244595.1
A. pollenicola	ATCC 62712	Canada	GCA_024244615.1

***Aspergillus* species**	**Strain/isolate designation**	**Origin**	**Genome / SRA run accession number**
Clinical isolates			
A. flavus	VF-40930/2022 (ASP1)	Internal collection, Institute of Microbiology and Parasitology, Veterinary faculty, University of Ljubljana, Slovenia	SRR36484474*
A. fumigatus	VF-41682/2022 (ASP2)	Internal collection, Institute of Microbiology and Parasitology, Veterinary faculty, University of Ljubljana, Slovenia	SRR36484473*
A. niger	VF-18763/2025 (ASP3)	Internal collection, Institute of Microbiology and Parasitology, Veterinary faculty, University of Ljubljana, Slovenia	SRR36484471*
Reference genomes			
A. flavus	NRRL 3357	USA	GCA_009017415.1
A. fumigatus	Af293	UK, human patient	GCA_000002655.1
A. niger	/	/	GCA_000002855.2

*sequenced in this study
