# Supplementary figures and images for: Rapid MALDI-TOF Mass Spectrometry Identification of the Chalkbrood Pathogen Ascosphaera apis
Source: J Fungi (Basel). 2026 Apr 23;12(5):311. doi: 10.3390/jof12050311 (PMC13208865; doi:10.3390/jof12050311)

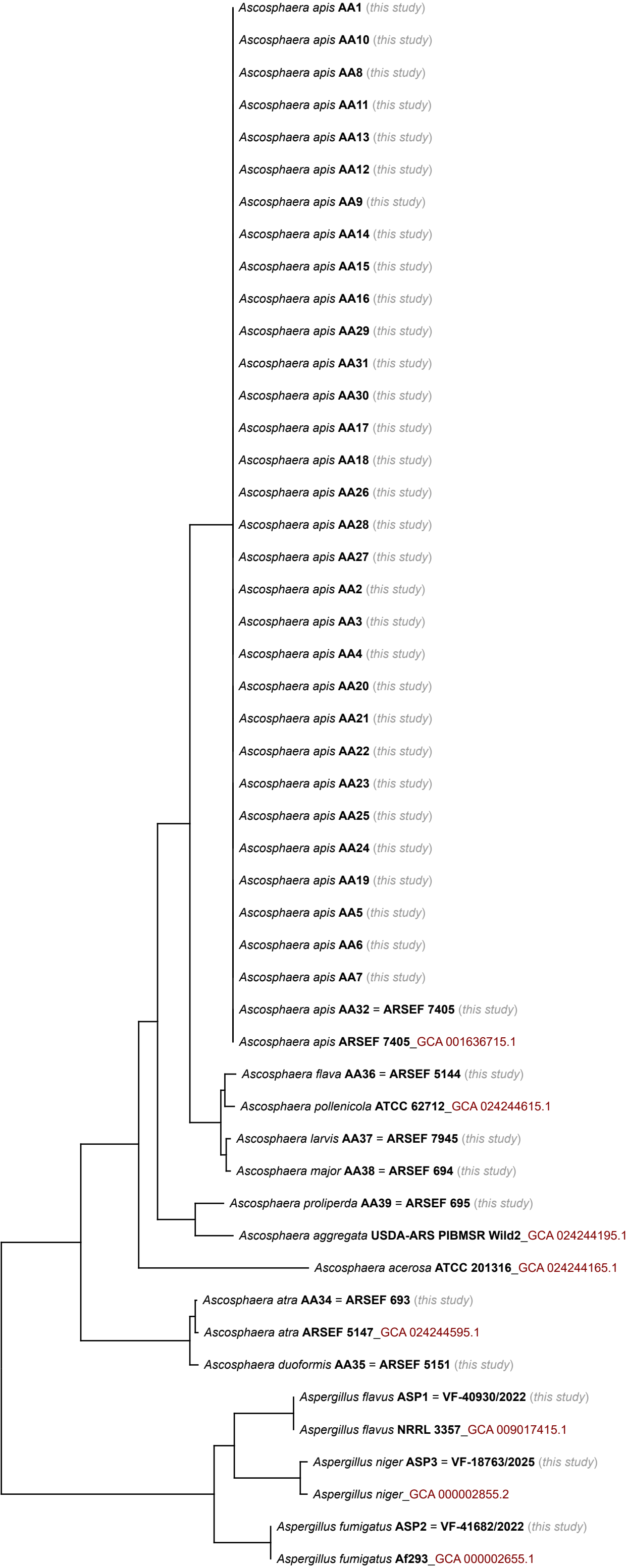

0.20

Supplement: Supplementary file 1 [file jof-12-00311-s001.zip › jof-4251818-supplementary/Hocevar et al_Figure_S1.pdf]
